# Anterior cruciate ligament remnant‐preserving and re‐tensioning reconstruction: a biomechanical comparison study of three different re‐tensioning methods in a porcine model

**DOI:** 10.1186/s12891-021-03955-w

**Published:** 2021-02-03

**Authors:** Dong Jin Ryu, Kyeu Back Kwon, Da Hee Hong, Sang Jun Park, Jae Sung Park, Joon Ho Wang

**Affiliations:** 1 Department of Orthopaedic Surgery, Inha University Hospital, Inha University School of Medicine, Incheon, South Korea; 2 Department of Orthopedic Surgery, Samsungbon Hospital, Osan, Korea; 3 Samsung Biomedical Research Institute, Samsung Medical Center, Sungkyunkwan University School of Medicine, Seoul, South Korea; 4 Department of Orthopaedic Surgery, Samsung Medical Center, Sungkyunkwan University School of Medicine, 06351 Seoul, South Korea; 5 Department of Health Sciences and Technology, SAIHST, Sungkyunkwan University, 06351 Seoul, South Korea; 6 Department of Medical Device Management and Research, SAIHST, Sungkyunkwan University, 06351 Seoul, South Korea

**Keywords:** ACL, Remnant-preserving, Re-tensioning, Biomechanical

## Abstract

**Background:**

With the developments in the arthroscopic technique, anterior cruciate ligament (ACL) remnant-preserving reconstruction is gradually gaining attention with respect to improving proprioception and enhancing early revascularization of the graft. To evaluate the mechanical pull-out strength of three different methods for remnant-preserving and re-tensioning reconstruction during ACL reconstruction.

**Methods:**

Twenty-seven fresh knees from mature pigs were used in this study. Each knee was dissected to isolate the femoral attachment of ACL and cut the attachment. An MTS tensile testing machine with dual-screw fixation clamp with 30° flexion angle was used. The 27 specimens were tested after applying re-tensioning sutures with No. 0 polydioxanone (PDS), using the single stitch (n = 9), loop stitch (n = 9), and triple stitch (n = 9) methods. We measured the mode of failure, defined as (1) ligament failure (longitudinal splitting of the remnant ACL) or (2) suture failure (tearing of the PDS stitch); load-to-failure strength; and stiffness for the three methods. Kruskal-Wallis test and Mann-Whitney U-test were used to compare the variance of load-to-failure strength and stiffness among the three groups.

**Results:**

Ligament failure occurred in all cases in the single stitch group and in all but one case in the triple stitch group. Suture failure occurred in all cases in the loop stitch group and in one case in the triple stitch group. The load-to-failure strength was significantly higher with loop stich (91.52 ± 8.19 N) and triple stitch (111.1 ± 18.15 N) than with single stitch (43.79 ± 11.54 N) (p = 0.002). With respect to stiffness, triple stitch (2.50 ± 0.37 N/mm) yielded significantly higher stiffness than the other methods (p = 0.001).

**Conclusions:**

The results suggested that loop stitch or triple stitch would be a better option for increasing the mechanical strength when applying remnant-preserving and re-tensioning reconstruction during ACL reconstruction.

## Background

Despite the recent successful outcomes of anterior cruciate ligament (ACL) reconstruction, the failure rate is still 8–25 % [1, 2]. For successful ACL reconstruction, various factors, including graft placement with firm fixation, incorporation, revascularization, and ligamentization, should be considered [2–4]. In addition to stability, good proprioceptive function is important [1, 4, 5]. Histologic studies of ACL remnant tissue have revealed the presence of mechanoreceptors and biological healing potential owing to vascular support by the synovial sheath [2, 4, 6]. With the developments in the arthroscopic technique, ACL remnant-preserving reconstruction is gradually gaining attention with respect to improving proprioception and enhancing early revascularization of the graft [1, 2, 4, 7].

The method of preserving the remnant fibers greatly varies from study to study, as follows: (1) merely leaving the tibial portion of the ACL stump [4, 8, 9], (2) selective-bundle augmentation in the presence of an abundant remnant bridging the femur and tibia [10, 11], and (3) femoral avulsion repair and augmentation with graft [1, 7, 12, 13]. However, preservation of the remnant ACL stump might lead to cyclops lesion formation, graft impingement, or incorrect tibial tunnel placement [1, 14]. The clinical results of the remnant-preserving method are still debated [2, 8].

Mechanoreceptors for proprioception can be stimulated by length changes, and the rate of changes in tension. [1, 15–18]. Furthermore, the strength of the remnant tissue in the early phase after reconstruction may be beneficial for rehabilitation and early incorporation [19]. Nagai et al. [20] reported that ACL remnants partially contributed to anterior–posterior stability. For these reasons, it would be better to provide a re-tensioning method to create a mechanically stable environment as possible [19].

Several methods of remnant-preserving and re-tensioning have been introduced, including (1) single stitch, (2) loop stitch, and (3) triple stitch [1, 7, 12, 13]. However, to our knowledge, there are no data on comparative outcomes of the ACL remnant-preserving and re-tensioning reconstruction technique in terms of mechanical strength. The aim of this study was to evaluate the time-zero mechanical pull-out strength with three different re-tensioning methods used in ACL reconstruction. We hypothesized that using the triple stitch method, rather than the simple stitch or loop stitch method, would result in better biomechanical outcomes.

## Methods

### Specimen preparation for the acute ACL complete femoral detachment model

**Fig. 1:**
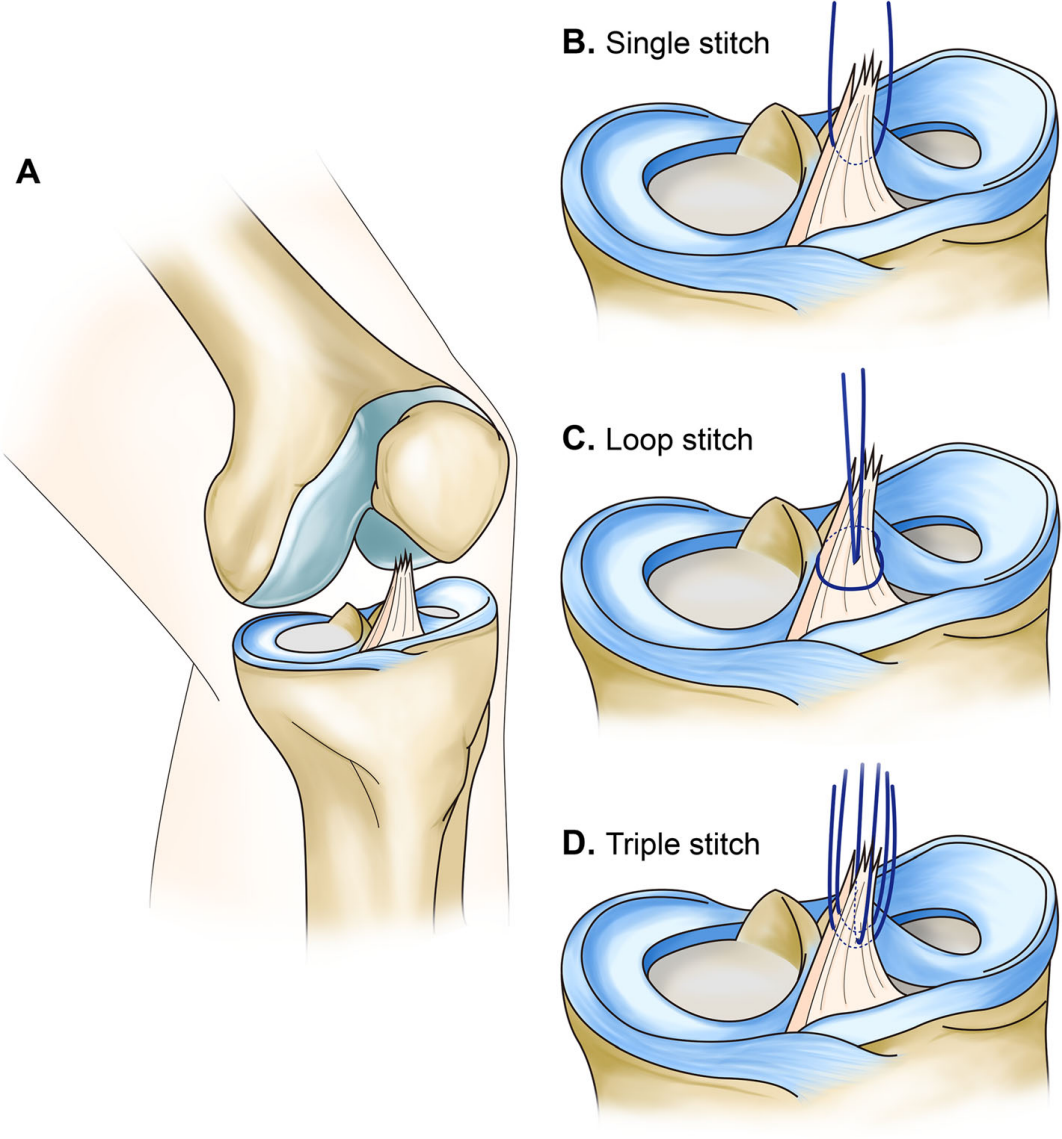
**a** Anterior cruciate ligament tear was made from the femoral-side insertion. Schematic drawing of each repair method: **b** Single stitch: passing one No. 0 polydioxanone (PDS) from the medial to lateral direction on the mid-substance of the ACL remnant, (**c**) Loop stitch: passing one suture loop through the mid-portion of the remnant, and the free ends of the suture were retrieved through the loop, and (**d**) Triple stitch: stitches were formed sequentially in the medial to lateral, anterior to posterior, and medial to lateral directions on the mid-substance portion of the remnant

Twenty-one fresh frozen knees from mature pigs (body weight, 127 ± 11.6 kg) were used in this study (Cellumed, Seoul, Republic of Korea). Each knee was dissected to remove the skin, muscle, collateral ligament, posterior cruciate ligament, medial and lateral meniscus, and patella. With any attachment of soft tissue between femur and tibia, after isolating the femoral attachment of ACL, we cut the ACL femoral attachment. We attempted to reproduce the ACL injury pattern (Type I or Type II, suggested by Sherman et al. [21]) as much as possible by cleanly transecting the ACL by three or four times from its femoral insertion site using a No. 11 blade (Fig. 1A). The remainder of the ACL, including the mid-substance and the tibial insertion site, was kept intact. The tibia bone was cut with an oscillating saw 7 cm below the knee joint line. All specimens were wrapped in gauze soaked in saline solution and stored at -4 °C until testing. Before the test day, the specimens were thawed for 6 h at 4 °C [7].

### Repair methods

To set the same condition, one type of suture material was used (No. 0 polydioxanone [PDS® II]). Single stitch was made by passing one PDS No. 0 from the medial to lateral direction on the mid-substance of the ACL remnant (Fig. 1B) [13]. Loop stitch was formed by passing one suture loop through the mid-portion of the remnant, and the free ends of the suture were retrieved through the loop [12] (Fig. 1C). Triple stitches were formed sequentially in the medial to lateral, anterior to posterior, and medial to lateral directions on the mid-substance portion of the remnant using three PDS No. 0 (Fig. 1D) [1].

### Load‐to‐failure test

**Fig. 2:**
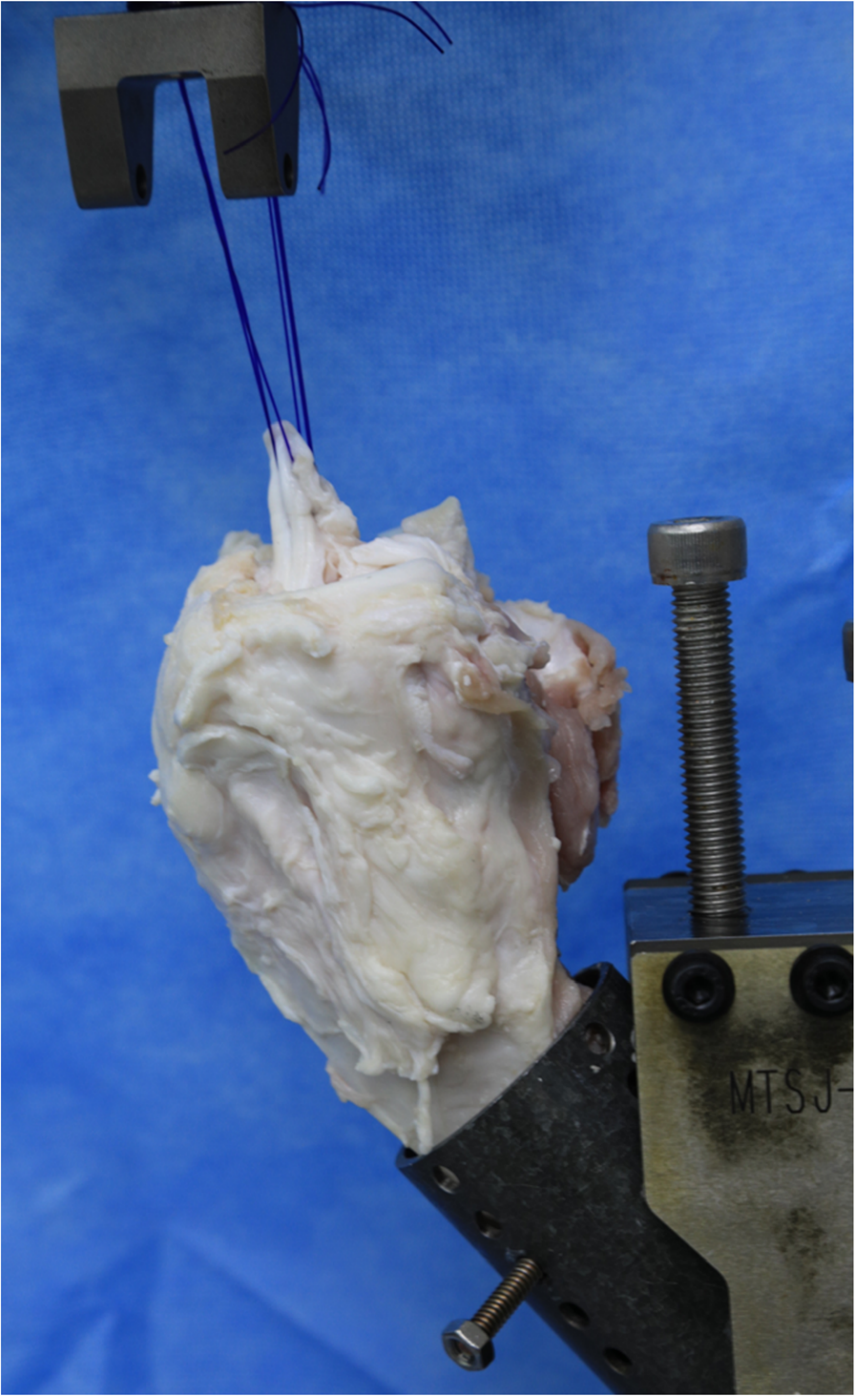
Tensile load-to-failure testing. The tibia is clamped to the MTS machine using dual-screw fixation with 30° flexion angle. The polydioxanone (PDS) suture was also tied and mounted on the custom-made device. The specimen was clamped in the testing fixture according to the instructions and tested at 30° of flexion angle. The clamped sample was adjusted so that the direction of the axial load was aligned with the long axis of the grafted tendon

The tensile test was performed on a tensile tester (858 Mini Bionix II; MTS Systems, Eden Prairie, MN). The tibia was fixed on a cylinder with dual-screw (6.0 mm) and nut fixation. Thereafter, the cylinder on which the tibia was fixed was mounted on a custom-made adjusting device. The PDS suture was also tied and mounted on the custom-made device. The specimen was clamped in the testing fixture according to the instructions and tested at 30° of flexion angle [7]. The clamped sample was adjusted so that the direction of the axial load was aligned with the long axis of the grafted tendon (Fig. 2).

A preload of 2 N was applied to the fixed specimens [7]. The tensile test was started at a crosshead speed of 5 mm/min [22]. The force resolution of the tensile tester was 0.01 N. A load-displacement curve was plotted. The ultimate load-to-failure and stiffness of the specimens were calculated. Stiffness was determined during load-to-failure testing from the maximal endpoint of the linear region of the load-displacement plot, and the relation between ultimate load and displacement was evaluated [7, 23]. The failure mode was determined through visual inspection and defined as (1) ligament failure (splitting of the remnant ACL) or (2) suture failure (tearing of the PDS stitch). Two experimental observers confirmed the failure mode [24, 25].

### Statistical analysis

Kruskal-Wallis test was used to compare the load-to-failure and stiffness among the three groups. The level of statistical significance was set at* p *< 0.05. The Mann-Whitney U-test was used to compare between-group differences assessed using Bonferroni’s adjustment for multiple testing (*p *< 0.05/3). On the basis of the data obtained in the first six specimens (2 for each group, total of 6), a sample size calculation (α = 0.05, β = 0.2) was conducted in terms of the mean and standard deviation of load-to-failure using G power 3.1 [26, 27]. A sample size of seven specimens in each group was calculated as the minimum requirement to ensure 80 % power for detecting differences in load-to-failure. Finally, a total of 21 knees evaluated. Statistical analysis was performed using SPSS software for Windows (version 25.0; SPSS, Chicago, IL).

## Results

### Mode of failure

**Fig. 3:**
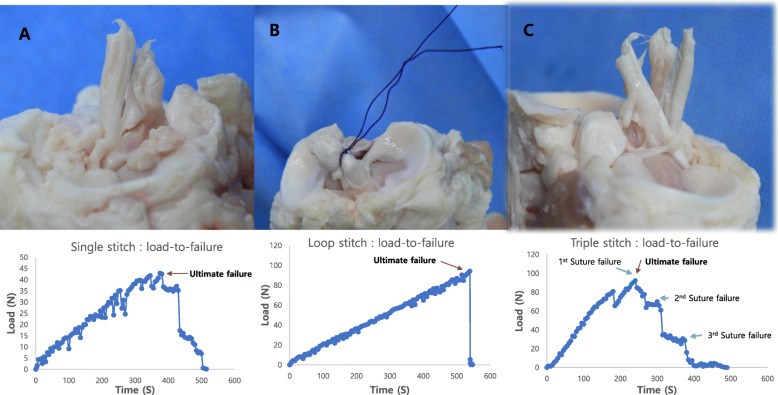
Mode of failure and representative load-to-failure graph for each method: (**a**) single stitch, (**b**) loop stitch, and (**c**) triple stitch *N: newtons, S: second

Ligament failure occurred in all cases in the single stitch group and in all but one case in the triple stitch group. Suture failure occurred in all cases in the loop stitch group and in one case in the triple stitch group. In the single stitch group, the load was dramatically decreased as mid-substance ligament failure occurred after reaching the ultimate load. As a result, a ligament longitudinal splitting failure pattern was observed in all cases (Fig. 3A). In the loop stitch group, failure occurred when the PDS suture was broken at approximately 90 N load (Fig. 3B). There was no injury in the ligament. In the triple stitch group, as the stitches failed one after another after reaching the ultimate failure, the load was stepwise decreased and the final failure occurred, resulting in the ACL fiber being split into three pieces (Fig. 3C).

### Load‐to‐failure and stiffness

**Fig. 4:**
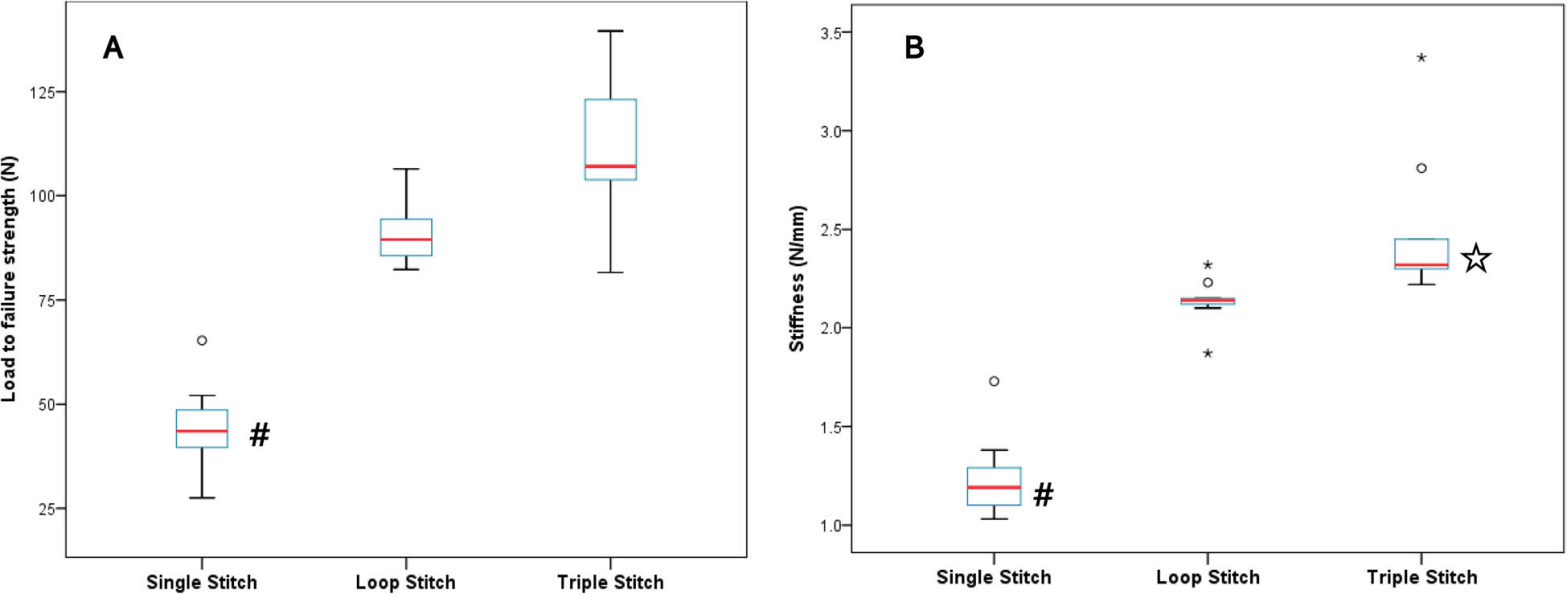
Box plot for mean load-to-failure strength and stiffness for each method (7 legs for each method). **a** Single stitch yielded significantly lower load-to-failure strength (#, *p *= 0.002). **b**Single stitch also yielded significantly lower stiffness (#, *p *< 0.001), whereas triple stitch yielded the highest stiffness among the three repair methods (☆, *p *= 0.001). †N: newton, error bar: range ‡statistical analysis : Kruskal-Wallis test with Bonferroni post hoc analysis

The load-to-failure strength was significantly higher with loop stitch (91.52 ± 8.19 N) or triple stitch (111.1 ± 18.15 N) than with single stitch (43.79 ± 11.54 N) (*p *= 0.002) (Fig. 4A). However, there was no significant difference between loop stitch and triple stitch (*p *= 0.18).

With respect to stiffness, there was a significant difference in the means of the three methods based on the Kruskal-Wallis test (*p *< 0.001). The Mann-Whitney U-test was again performed for pairwise comparisons. The triple stitch group had the highest stiffness (2.50 ± 0.37 N/mm), closely followed by the loop stitch group (2.13 ± 0.12 N/mm) and finally by the single stitch group (1.24 ± 0.22 N/mm) (Fig. 4B). The triple stitch method yielded significantly higher stiffness than the loop stitch method (*p *= 0.001) and the single stitch method (*p *< 0.001).

## Discussion

The most important finding of this study was that the loop stitch and triple stitch methods provided higher time-zero load-to-failure strength. Moreover, the triple stitch method yielded the highest stiffness among the three re-tensioning methods. Thus, our hypothesis was proved to be correct. The failure pattern was observed to differ according to the repair method. As a result, the single and triple stitch groups had longitudinal tissue splitting patterns, and all cases in the loop stitch group failed at approximately 90 N, which is similar to the ultimate load-to-failure strength of PDS No. 0 [28]. The single and triple stitch methods have the advantage of being technically easy and simple to perform; however, if excessive force is applied during re-tensioning, there is a risk of additional damage to the ACL remnant fibers. Further, the triple stich method may be difficult to apply depending on the remnant tissue quality and status [29] With respect to the loop stitch method, better wrapping and coverage around the graft can be achieved [12], but a single stitch could not yield sufficient strength. Therefore, adding a single simple stitch to the loop stitch could result in greater mechanical strength. The disadvantage of the loop stitch method is that it is technically demanding and time consuming through the arthroscopy procedure, and additional equipment (Knee Scorpion; Arthrex, Naples, FL) would be required to make it more convenient to use.

Theoretically, as in posterior cruciate ligament reconstruction, augmentation with remnant tissue might be helpful for biological healing in ACL reconstruction [1]. Moreover, remnant tissue helps in revascularizing the graft, incorporating the graft [30], preserving proprioception [15, 19], reducing tibial tunnel widening [8], reducing synovial fluid leakage [13], and mechanically protecting the graft from being scratched by the intercondylar notch [13]. The preserved proprioceptive nerve fibers would reinnervate the reconstructed ACL [4]. On the other hand, some studies showed no difference between “remnant-preserving” and “conventional single-bundle” ACL reconstruction [8, 31]. However, the advantages of remnant preservation are difficult to reveal using clinical scores or physical examination [1, 15]. Instead, they can be proved by the long-term failure rate or proprioceptive function. However, despite the technological advances, there are still many limitations in accurately assessing proprioceptive function [15, 32].

Some of the previous ACL reconstruction procedures for which the term “remnant-preserving technique” was used were performed with type 4 ACL remnant tissue as categorized by Crain et al. [29] The method used in previous studies was not much different from single-bundle reconstruction, without repair or re-tensioning and merely leaving fibers on the tibial footprint [4, 8, 9]. Many of these studies reported no significant difference between remnant-preserving and conventional single-bundle reconstruction. However, for remnant tissue to play a role and properly function, (1) good quality of the remnant and (2) appropriate tension are necessary. Leaving only some remnant of the tibial side may have a synergic effect on graft incorporation, synovialization, and revascularization; however, it is unlikely to significantly affect the stability and proprioception [15, 17, 19]. Rather, the risk of cyclops lesion could be increased [2, 19].

Longitudinal tension on a ligament results in compression of the connective tissue, leading to stimulation of the mechanoreceptors. Mechanoreceptors can also be stimulated by length changes, as well as by the rate of changes in tension and length [15, 17, 18, 33]. Therefore, it is necessary to create an environment in which mechanoreceptors can be adequately stimulated. Accordingly, “repair or re-tensioning with graft augmentation” may be a more appropriate method for effective maintenance and recovery of proprioception than simply “conserving” the remnant.

In this study, only the mechanical strength of the remnant fiber was assessed. In practice, however, as autogenic or allogenic graft tendons are used as the main ACL graft, the strength of remnant fibers is relatively insignificant. However, with the concept of additional fibers, it is reasonable to apply a method that can withstand as much force as possible [16, 19]. Further studies are needed to compare the mechanical strength of the single-bundle method alone and single bundle augmentation with remnant repair method.

This study has several limitations. First, the results were based on an in vitro biomechanical study and time-zero results and did not account for biological factors such as ligament incorporation, collateral ligament, posterior cruciate ligament, or the effect of the meniscus as a knee co-stabilizer. Also, we examined without cyclic loading. The compromised ACL is difficult to tolerate cyclic load by itself and is used as a concept of augmentation on the graft tendon. Thus, in previous studies about ACL remnant strength, only preload was applied and cyclic load was not applied [7, 22]. Therefore, in this study, only axial loading was performed to test with protecting of ACL remnants. Second, as previously noted, this study used porcine knees, which may not entirely correspond to human knees. Nonetheless, the key anatomic features and functional characteristics of the porcine model are similar to those of human knees [34]. Moreover, porcine models are commonly used in biomechanical studies of the meniscus [27, 35]. Third, we measured the mechanical strength with tensile loading at 30° flexion [7]; however, the actual human knee moves dynamically through a wide range of angles, rotation, and pivoting, and the axial tensile load used in this study might not reflect the behavior of the knee during real-life functional activities. Fourth, to maximally simulate real conditions, re-tensioning was applied after cutting the ACL at the femoral attachment. There is a possibility that the state of the surrounding soft tissues, such as synovium adhesion, may be different for each experimental subject. This may have affected the load-to-failure and stiffness measurements. Fifth, what we created was a complete femoral ACL “detachment” model, not a real “rupture” model. This issue should be taken into account because differences may exist between these two different injury patterns in terms of healing capacity [7, 36]. Sixth, we could not simulate actual condition of ACL reconstruction. During the ACL reconstruction procedure, the tibial side ACL remnant would be impaired. As introduced by Ahn et al. [1] and Noh et al. [37] with medial traction of the remnant using sutures and with final hand reaming technique could reduce additional injury of remnant ACL tibia foot print. Seventh, the suture material used in repair was No. 0 PDS. Each surgeon may prefer a different material (absorbable vs. non-absorbable) [1, 7, 12, 13] and the results may vary depending on the suture material used.

## Conclusions

The results of this study suggest that loop stitch or triple stitch would be a better option for increasing the mechanical strength when applying remnant-preserving and re-tensioning reconstruction during ACL reconstruction.

## Data Availability

The datasets used and/or analyzed during the present study are available from the corresponding author on reasonable request.

## Abbreviations

ACL: Anterior cruciate ligament
PDS: Polydioxanone suture

## References

[1] Ahn JH, Wang JH, Lee YS, Kim JG, Kang JH, Koh KH (2011). Anterior cruciate ligament reconstruction using remnant preservation and a femoral tensioning technique: clinical and magnetic resonance imaging results. Arthroscopy.

[2] Song G-Y, Zhang H, Zhang J, Li X, Chen X-Z, Li Y (2013). The anterior cruciate ligament remnant: to leave it or not?. Arthroscopy: J Arthrosc Related Surg.

[3] Bach BR, Tradonsky S, Bojchuk J, Levy ME, Bush-Joseph CA, Khan NH (1998). Arthroscopically assisted anterior cruciate ligament reconstruction using patellar tendon autograft. Five- to nine-year follow-up evaluation. Am J Sports Med.

[4] Takazawa Y, Ikeda H, Kawasaki T, Ishijima M, Kubota M, Saita Y (2013). ACL reconstruction preserving the ACL remnant achieves good clinical outcomes and can reduce subsequent graft rupture. Orthopaedic J Sports Med.

[5] Noyes FR, Butler DL, Paulos LE, Grood ES. Intra-articular cruciate reconstruction. I: Perspectives on graft strength, vascularization, and immediate motion after replacement. Clin Orthop Relat Res. 1983;172:71–7. 6337002

[6] Dhillon MS, Bali K, Vasistha RK (2010). Immunohistological evaluation of proprioceptive potential of the residual stump of injured anterior cruciate ligaments (ACL). Int Orthop.

[7] Song G, Zhang J, Li X, Li Y, Feng H (2016). Biomechanical and biological findings between acute anterior cruciate ligament reconstruction with and without an augmented remnant repair: a comparative in vivo animal study. Arthroscopy:&nbsp;J Arthrosc Relat Surg.

[8] Zhang Q, Zhang S, Cao X, Liu L, Liu Y, Li R (2014). The effect of remnant preservation on tibial tunnel enlargement in ACL reconstruction with hamstring autograft: a prospective randomized controlled trial. Knee Surg Sports Traumatol Arthrosc..

[9] Kim MK, Lee SR, Ha JK, Ra HJ, Kim SB, Kim JG (2014). Comparison of second-look arthroscopic findings and clinical results according to the amount of preserved remnant in anterior cruciate ligament reconstruction. Knee.

[10] Ochi M, Adachi N, Deie M, Kanaya A (2006). Anterior cruciate ligament augmentation procedure with a 1-incision technique: anteromedial bundle or posterolateral bundle reconstruction. Arthroscopy.

[11] Ochi M, Adachi N, Uchio Y, Deie M, Kumahashi N, Ishikawa M (2009). A minimum 2-year follow-up after selective anteromedial or posterolateral bundle anterior cruciate ligament reconstruction. Arthroscopy.

[12] Boutsiadis A, Karampalis C, Tzavelas A, Vraggalas V, Christodoulou P, Bisbinas I (2015). Anterior cruciate ligament remnant–preserving reconstruction using a “Lasso-Loop” knot configuration. Arthrosc Techn.

[13] Noh JH, Kyung HS, Roh YH, Kang TS (2017). Remnant-preserving and re-tensioning technique to cover the graft in anterior cruciate ligament reconstruction. Knee Surg Sports Traumatol Arthrosc..

[14] Jackson DW, Schaefer RK (1990). Cyclops syndrome: loss of extension following intra-articular anterior cruciate ligament reconstruction. Arthroscopy.

[15] Nakase J, Toratani T, Kosaka M, Ohashi Y, Tsuchiya H (2013). Roles of ACL remnants in knee stability. Knee Surg Sports Traumatol Arthrosc.

[16] Kosy J, Mandalia V (2018). Anterior cruciate ligament mechanoreceptors and their potential importance in remnant-preserving reconstruction: a review of basic science and clinical findings. J Knee Surg.

[17] van der Wal J (2009). The architecture of the connective tissue in the musculoskeletal system-an often overlooked functional parameter as to proprioception in the locomotor apparatus. Int J Ther Massage Bodywork.

[18] Johansson H, Sjölander P, Sojka P (1991). Receptors in the knee joint ligaments and their role in the biomechanics of the joint. Crit Rev Biomed Eng.

[19] Muneta T, Koga H (2017). Anterior cruciate ligament remnant and its values for preservation. Asia-Pac J Sports Med Arthrosc Rehabil Technol.

[20] Nagai K, Araki D, Matsushita T, Nishizawa Y, Hoshino Y, Matsumoto T (2016). Biomechanical function of anterior cruciate ligament remnants: quantitative measurement with a 3-Dimensional electromagnetic measurement system. Arthroscopy.

[21] Sherman MF, Lieber L, Bonamo JR, Podesta L, Reiter I (1991). The long-term followup of primary anterior cruciate ligament repair. Defining a rationale for augmentation. Am J Sports Med.

[22] Zhang L, Jiang K, Chai H, Zhou M, Bai J (2016). A comparative animal study of tendon grafts healing after remnant-preserving versus conventional anterior cruciate ligament reconstruction. Med Sci Monit.

[23] Mitchell R, Pitts R, Kim Y-M, Matava MJ (2016). Medial meniscal root avulsion: A biomechanical comparison of 4 different repair constructs. Arthroscopy.

[24] Razek A, Fouda KA, Elmetwaley NS, Elbogdady NE (2009). Sonography of the knee joint. J Ultrasound.

[25] Razek AAKA, El-Basyouni SR (2016). Ultrasound of knee osteoarthritis: interobserver agreement and correlation with Western Ontario and McMaster Universities Osteoarthritis. Clin Rheumatol.

[26] Faul F, Erdfelder E, Buchner A, Lang A-G (2009). Statistical power analyses using G*Power 3.1: Tests for correlation and regression analyses. Behav Res Methods.

[27] Chung KS, Choi CH, Bae TS, Ha JK, Jun DJ, Wang JH (2018). Comparison of tibiofemoral contact mechanics after various transtibial and all-inside fixation techniques for medial meniscus posterior root radial tears in a porcine model. Arthroscopy: J Arthrosc Relat Surg.

[28] Kreszinger M, Toholj B, Ačanski A, Balos S, Cincović M, Pećin M (2018). Tensile strength retention of resorptive suture materials applied in the stomach wall - An in vitro study. Veterinarski arhiv.

[29] Crain EH, Fithian DC, Paxton EW, Luetzow WF (2005). Variation in anterior cruciate ligament scar pattern: does the scar pattern affect anterior laxity in anterior cruciate ligament-deficient knees?. Arthroscopy.

[30] Claes S, Verdonk P, Forsyth R, Bellemans J (2011). The “ligamentization” process in anterior cruciate ligament reconstruction: what happens to the human graft? A systematic review of the literature. Am J Sports Med.

[31] Hong L, Li X, Zhang H, Liu X, Zhang J, Shen JW (2012). Anterior cruciate ligament reconstruction with remnant preservation: a prospective, randomized controlled study. Am J Sports Med.

[32] Han J, Waddington G, Adams R, Anson J, Liu Y (2016). Assessing proprioception: A critical review of methods. J Sport Health Sci.

[33] Michelson JD, Hutchins C (1995). Mechanoreceptors in human ankle ligaments. J Bone Joint Surg Br.

[34] Dye SF (1987). An evolutionary perspective of the knee. J Bone Joint Surg Am.

[35] Cone SG, Lambeth EP, Ru H, Fordham LA, Piedrahita JA, Spang JT (2019). Biomechanical function and size of the anteromedial and posterolateral bundles of the ACL change differently with skeletal growth in the pig model. Clin Orthop Relat Res.

[36] Song G-Y, Zhang J, Li X, Chen X-Z, Li Y, Feng H (2014). Acute anterior cruciate ligament reconstruction with an augmented remnant repair: a comparative macroscopic and biomechanical study in an animal model. Arthroscopy.

[37] Noh JH, Yoon KH, Song SJ, Roh YH (2014). Re-tensioning technique to cover the graft with remnant in anterior cruciate ligament reconstruction. Arthrosc Tech.

